# Prevalence of Yeasts in English Full Blood Mares

**DOI:** 10.1007/s11046-013-9615-6

**Published:** 2013-01-24

**Authors:** Paweł Różański, Brygida Ślaska, Dorota Różańska

**Affiliations:** 1 Department of Animal and Environmental Hygiene, Faculty of Animal Breeding and Biology, University of Life Sciences in Lublin, Akademicka 13, 20-950 Lublin, Poland; 2 Department of Biological Bases of Animal Production, Faculty of Animal Breeding and Biology, University of Life Sciences in Lublin, Akademicka 13, 20-950 Lublin, Poland; 3 Department and Clinic of Animal Surgery, Faculty of Veterinary Medicine, University of Life Sciences in Lublin, Akademicka 13, 20-950 Lublin, Poland

**Keywords:** Horses, Fungi, Qualitative analysis

## Abstract

The aim of the study was a quantitative and qualitative analysis of microflora, presentation of current data about prevalence of the microflora on the skin and mucous membranes, and determination of its possible effect on reproduction of English full blood horses bred in Poland. The material for analyses was sampled from the skin and mucous membranes (385 samples) of 55 English full blood mares. Taking into account reproduction traits, the mares were classified into three groups. Six yeast-like species, including five species from the genus *Candida* (*C. albicans*, *C. guiliermondii*, *C. lusitaniae*, *C.* sp., and *C. tropicalis*) and *Trichomonascus ciferrii*, were detected on the skin and mucous membranes in the English full blood mares. Growth of yeasts was observed in more than half of the samples taken from mares that had foaled and approximately 46 % of non-conceiving and barren mares. The high prevalence of various yeast strains in the mouth, nostrils, and collateral groove may suggest widespread occurrence of the microflora in the breeding environment. The results obtained indicate that the yeasts isolated in this study may be components of the normal microflora of the skin and mucous membranes in horses. The analysis results do not indicate unambiguously that the isolated microflora affects reproduction in mares, although this cannot be excluded.

## Introduction

Microorganisms are responsible for development of many diseases, which directly or indirectly affect reproductive performance in various animal species. Knowledge of the microflora of animal skin and mucous membranes is essential for better understanding of pathological processes. Few publications have described prevalence of normal fungal flora in the animal reproductive tract. Some fungi are potentially pathogenic. According to Garoussi et al. [1], in cows, these include *Candida albicans* (39.1 %), *Geotrichum* (8.7 %), *Cr. neoformans* (4.3 %), and other yeasts (13 %). In horses, pathogenic fungi are *Candida albicans, Candida parapsilosis, Candida lusitaniae, Candida rugosa, Cr. neoformans, Hansenula anomala, Hansenula polymorpha, Rhodotorula minuta, Rhodotorula rubra*, and *Torulopsis candida* [2], and in dogs, according to Cleff et al. [3], *C. parapsilosis* (21.7 %), *Candida guilliermondi* (8.4 %), *Candida kefyr* (6 %), and *Candida albicans* (4.8 %).

Previously, not much attention was paid to fungal infections of the reproductive tract in animals. However, extensive antibioticsand hormonal therapies have resulted in rising incidence of fungal infections in humans [4]. Verma et al. [5] demonstrated that various fungi, including *Aspergillus, Penicillium, Acremonium, Cladosporium, Mortierella, Aureobasidium pullulans, Zygomycetes*, and *Candida,* might cause infections of the reproductive organs in cows and buffalos. Vaginal, uterine, and cervical inflammations caused by fungal infections have also been reported from horses [6, 7], cat [8], and dogs [3].

The microflora present in the breeding environment of various groups of healthy horses was described by Różański et al. [9]. The 13 species (8 of which belonged to the genus *Candida*) comprised only yeast-like opportunistic microorganisms that are regarded as invasive species by other authors, for example, *C. albicans*, *Candida glabrata*, *Candida tropicalis*, *Candida krusei, C. guilliermondi, C. rugosa*, and *Trichomonascus ciferrii*.

Given the potential causative role of yeast-like fungi in endogenous infections, identification of fungal flora occurring on the skin and mucous membranes of English full blood horses will contribute to elucidation of the role of these microorganisms in various types of infections, including asymptomatic infections of reproductive organs. Furthermore, this investigation will be helpful in determination of the species composition of the physiological yeast-like microflora occurring on the skin and mucous membranes in English full blood horses.

The aim of the study was a quantitative and qualitative analysis of microflora, presentation of current data about prevalence of the microflora on the skin and mucous membranes, and determination of its possible effect on reproduction of English full blood horses bred in Poland.

## Materials and Methods

The investigations were carried out on material sampled from 55 English full blood mares aged between 4 and 12. All the horses were kept in separate boxes, in two brick, metal-roofed stables in one of the equestrian centres in Poland.

The mares were classified into three groups, where group I comprised 12 high-quality mares kept in the boxes together with their offspring in stable B. Group II consisted of mares that had not conceived after mating. These were 24 mares, which had previously had healthy foals, but during the last 2 seasons failed to conceive; they were kept in separate boxes in stable A. Group III was composed of 19 barren mares that had not foaled within three or more years and were excluded from reproduction (stable B). It should be stressed that the examinations involved all mares with reproductive disturbances. Mares that had foaled at an approximate time were randomly chosen for analyses from a bigger group of mares in the stud.

Temperature was measured with an instant-read liquid thermometer. Relative humidity was assessed using the Assmann’s aspiration psychrometer. The mean values of temperature and relative humidity were, respectively, 23 °C and 54 % outside, and 21 °C and 76 % inside the boxes.

Smears from nostrils, mouth, ear, back, groin, vagina, and collateral groove were taken from each mare. Material obtained upon inoculation of 385 samples was analysed. The samples were collected once in the summer period (July).

All the samples were inoculated on the Sabouraud medium; next, macroscopically homogenous colonies were obtained in selective cultures, which, after growing, were assayed with the API 20C AUX (bioMérieux) biochemical tests. The ability of biofilm formation was also assayed.

All the horses kept in the stables were fed in accordance with the current horse feeding standards (Horse Feeding Standards. Collaborative work, Institute of Physiology and Animal Nutrition, Polish Academy of Sciences, Jabłonna 1997) providing mineral-vitamin supplements.

The experimental horses received permanent zootechnical and veterinary care. All the animals underwent a prophylaxis programme of parasitic invasion control and vaccinations. All the horses were healthy during the investigations. None of them received any treatment before (at least 1 month earlier) or after the study (within minimum 1 month).

## Results

The greatest abundance of yeast fungi was found in the smear samples from mouths, nostrils, and collateral grooves (78.8, 64.4, and 68.0 %, respectively) in all the mares. The samples collected from the vaginal mucous membrane and groin skin contained the smallest number of the microorganisms (14.4 and 18.9 %, respectively).

Representatives of six microbial species, including five species of the genus *Candida*, were detected on the skin and mucous membranes of the experimental mares (Table 1). Growth of yeasts was detected in over a half of the samples taken from mares that had foaled and in ca. 46 % of the samples collected from mares that had not conceived during previous oestrus and mares that had been barren for minimum 3 seasons. Three species were found in each group. The smallest number of species was identified on the skin and mucous membranes of mares that had foaled. There were five species isolated in the samples from each of the other two mare groups (Table 1). Two species, that is *C. albicans,* isolated from the mares that had not conceived during the previous oestrus, and *C. lusitaniae*, isolated from the same group and mares that had been barren for minimum 3 seasons were not detected in the mares that had foaled. None of the 182 isolated yeast strains exhibited the ability to form biofilm.

**Table 1:** Frequency of yeast species isolated from the skin and mucous membranes of English full blood mares in relation to the reproduction performance

Isolated strains	Group I no. (%)	Group II no. (%)	Group III no. (%)
*C. albicans*	–	2 (1.2)	–
*C. guiliermondii*	25 (29.8)	58 (34.5)	32 (24.1)
*C. lusitaniae*	–	4 (2.4)	8 (6.0)
*C.* sp.	6 (7.1)	7 (4.2)	4 (3.0)
*C. tropicalis*	12 (14.3)	–	16 (12.0)
*T. ciferrii*	1 (1.2)	6 (3.6)	1 (0.8)
No-growth samples	40 (47.6)	91 (54.2)	72 (54.1)
Number of samples	84 (100.0)	168 (100.0)	133 (100.0)

Growth of yeasts was detected in more than 52 % of the samples collected from the mares that had foaled (Table 2). Yeast-like flora was isolated from the vagina and groin in the smallest number of the mares. *C. tropicalis* was found in the vagina of one mare only. Various microbial species were detected on the mouth and nostril mucosa in 92 and 75 % of the mares, respectively (Table 2). The most common yeast in mares that had foaled was *C. guiliermondii*, which was not found on the vaginal mucosa only.

**Table 2:** Frequency of yeast species isolated from the skin or mucous membranes of English full blood mares that had foaled (Group I)

Isolated strains	Nostrils	Mouth	Ear	Back	Groin	Vagina	Collateral groove
*C. guiliermondii*	4 (33.3)	6 (50.0)	2 (16.7)	4 (33.3)	3 (25.0)	0 (0.0)	6 (50.0)
*C.* sp.	2 (16.7)	2 (16.7)	0 (0.0)	1 (8.3)	0 (0.0)	0 (0.0)	1 (8.3)
*C. tropicalis*	2 (16.7)	3 (25.0)	3 (25.0)	2 (16.7)	0 (0.0)	1 (8.3)	1 (8.3)
*T. ciferrii*	1 (8.3)	0 (0.0)	0 (0.0)	0 (0.0)	0 (0.0)	0 (0.0)	0 (0.0)
No growth	3 (25.0)	1 (8.3)	7 (58.3)	5 (41.6)	9 (75.0)	11 (91.7)	4 (33.3)

No. (Frequency)—applies to Tables 2, 3, 4

Among the seven sample collection sites in the mares with conception disorders, the biggest numbers of yeasts were isolated from the smears taken from the back skin and nostril mucosa (in 71 and 67 %, respectively). The number of isolates obtained from other sites was smaller; they were detected in 21–64 % of the samples. The smallest number of strains was isolated from the groin (12.6 %) and vaginal (21 %) material.

In group II, *C. guiliermondii* was most prevalent and constituted over 75 % of all microorganisms identified in the mares with conception failure.

Yeast growth was detected in 46 % of the samples taken from the barren mares (Table 4). The highest growth was found in the mouth and collateral groove (89 and 79 %, respectively). Yeast growth on the vaginal mucosa was noted only in 11 % of the samples. The species identified in those sites were *C. guiliermondii* and *C. tropicalis*, which were the most prevalent yeasts in the group of the barren mares and constituted 52 and 26 %, respectively, of all the isolated yeasts (Table 4).

## Discussion

This paper presents pioneer study results. Available literature does not provide information about prevalence and composition of yeast-like microflora on the skin and mucous membranes of English full blood horses. Such data may confirm or exclude the relationship between the yeast colonizing the horse’s organism and reproductive failure. In order to determine the above-mentioned correlations, knowledge of quantitative and qualitative characteristics of the normal microflora occurring on the horse skin and mucous membranes is indispensable.

The greatest abundance of various yeast strains indentified in the mouth, nostrils, and collateral groove in the horses (Tables 2, 3, 4) may indicate widespread prevalence of microflora in the breeding environment. Analyses of the microflora in the living environment of several groups of healthy horses were performed and its prevalence described by Różański et al. [9]. The authors identified 13 species of various yeasts in the breeding environment of five horse groups, including four species present in the environment of English full blood horses (*C. guiliermondii*, *C. tropicalis*, *C.* sp., and *T. ciferrii*). Interestingly, the microbial presence in the test material was not accompanied by any deleterious health effects in the animals. The representatives of the fungal microflora were natural components of the environmental flora [9].

**Table 3:** Frequency of yeast species isolated from the skin or mucous membranes of English full blood mares with reproductive conception disorders (Group II)

Isolated strains	Nostrils	Mouth	Ear	Back	Groin	Vagina	Collateral groove
*C. albicans*	0 (0.0)	2 (8.4)	0 (0.0)	0 (0.0)	0 (0.0)	0 (0.0)	0 (0.0)
*C. guiliermondii*	13 (54.1)	12 (50.0)	3 (12.5)	13 (54.1)	2 (8.4)	4 (16.8)	11 (45.9)
*C. lusitaniae*	0 (0.0)	0 (0.0)	2 (8.4)	2 (8.4)	0 (0.0)	0 (0.0)	0 (0.0)
*C.* sp.	0 (0.0)	1 (4.2)	1 (4.2)	0 (0.0)	1 (4.2)	1 (4.2)	3 (12.5)
*T. ciferrii*	3 (12.5)	0 (0.0)	1 (4.2)	2 (8.4)	0 (0.0)	0 (0.0)	0 (0.0)
No growth	8 (33.4)	9 (37.4)	17 (70.7)	7 (29.1)	21 (87.4)	19 (79.0)	10 (41.5)

**Table 4:** Frequency of yeast species isolated from the skin or mucous membranes of English full blood barren mares (Group III)

Isolated strains	Nostrils	Mouth	Ear	Back	Groin	Vagina	Collateral groove
*C. guiliermondii*	9 (47.4)	7 (36.8)	1 (5.3)	3 (15.9)	2 (10.6)	1 (5.3)	9 (47.4)
*C. lusitaniae*	0 (0.0)	3 (15.9)	2 (10.6)	2 (10.6)	1 (5.3)	0 (0.0)	0 (0.0)
*C.* sp.	0 (0.0)	1 (5.3)	0 (0.0)	1 (5.3)	1 (5.3)	0 (0.0)	1 (5.3)
*C. tropicalis*	1 (5.3)	5 (26.3)	3 (15.9)	1 (5.3)	0 (0.0)	1 (5.3)	5 (26.3)
*T. ciferrii*	0 (0.0)	1 (5.3)	0 (0.0)	0 (0.0)	0 (0.0)	0 (0.0)	0 (0.0)
No growth	9 (47.4)	2 (10.6)	13 (68.4)	12 (63.1)	15 (78.9)	17 (89.4)	4 (21.2)

It is noteworthy that the yeast growth in the samples collected from the vagina was low in all the horse groups (from 8 to 21 % of all the samples analysed). Yet, it was twofold higher in mares with reproductive failure than in the foaling and barren mares. This may lead to a conclusion that a correlation between the presence of yeasts in the vagina and reproduction performance cannot be ruled out; 80 % of the microflora isolated from the vaginal mucosa of the mares with conception failure were *C. guiliermondii*, which was the most common yeast in the mares from all the groups (Table 1). These yeasts were not detected in the vaginas of the mares that had foaled.

Pfaller and Diekema [10] found 3–5 % serious invasive *Candida*-associated fungal infections to be caused by several different species, for example *C. guilliermondii* or *C. lusitaniae*. Chengappa et al. [2] reported that *C. guilliermondii* was one of the pathogenic yeast species. It was described as a causative agent of cervical, uterine, and vaginal infections in mares. The results obtained in this investigation and data provided by the literature cited imply a possible relation between *C. guilliermondii* occurrence and reproductive failure in English full blood mares.

Studies carried out by Ślaska et al. [11] exploring the microflora in Hucul Horses demonstrated existence of various strains within the *C. rugosa* species. It should be mentioned that genetic differences may exist also within the *C. guilliermondii* species, depending on its occurrence site. This assumption, however, needs further investigations at the molecular level.

There are diverse factors responsible for animal infertility, including mares. In this study, we proved that 79 % of mares with reproductive conception disorders show no growth of yeast species from vaginal samples (Tab. 3). However, from 16,8 % of mares from this group, there was isolated *C. guiliermondii*. It can be assumed that only *C. guiliermondii* may be indirectly associated with reproductive failure. *C. tropicalis* may be regarded as opportunistic microflora in mares, which is corroborated by its presence in the vaginas of mares that have foaled (Tables 1, 2). In contrast, this species has been detected in patients with haematological malignancies [10]. In mares, these yeasts have been described as the causative agent of reproductive tract infections [2].

Two species, which were not detected in mares that had foaled, are of special interest: *C. albicans* isolated from non-conceiving mares and *C. lusitaniae*—from non-conceiving and barren animals (Tables 3, 4). Since these species did not occur on the vaginal mucosa in mares with conception failure and barren mares, the species cannot be directly regarded as causative agents of reproductive failure. Their occurrence may seem peculiar, as the experimental horses did not exhibit any disease symptoms.

Diseases caused by both yeast and mould fungi are often reported to be pathogenic to humans and various animal species [5, 6, 10, 12]. Blue [6] found that fungal inflammation of the reproductive tract in mares was caused by *Aspergillus fumigatus* and *C. albicans* infections.

Besides *C. rugosa* and *C. krusei*, *C. albicans* is one of the most frequently isolated yeasts causing *mycotic mastitis* in cattle [12]. Reilly and Palmer [13] described foals with birth hypoxia (renal failure and necrotizing enterocolitis) and septicaemia during the first 2 days of life. They were diagnosed with systemic candidiasis after *C. albicans* had been isolated from one or several internal sites. Ledbetter et al. [14] examined horses with clinical signs of keratomycosis. The fungi that were most frequently isolated included *Aspergillus*, *Candida*, and *Fusarium* spp. Cases of equine infectious arthritis caused by *Candida* spp. have also been reported [15]. *Candida albicans* has been reported to be the most prevalent agent of candidiasis in humans. Yeast species from the genus *Candida* (*C. albicans, C. parapsilosis, C. glabrata,* and *C. tropicalis*) are regarded as the predominant etiological disease factors worldwide [16]. Similarly, Pfaller and Diekema [10] reported that severe invasive *Candida*-associated fungal infections in humans were caused by several species, for example *C. albicans*, *C. tropicalis C. guilliermondii,* and *C. lusitaniae*. All these species were isolated from the skin and mucous membranes of the English full blood horses, which showed no signs of disease (Tables 2, 3, 4).

Another interesting yeast species is *T. ciferrii* isolated from the skin and mucous membranes. It was reported to be the causative agent of human invasive external and internal mycosis [17]. *T. ciferrii* is remarkable, as it tends to acquire drug resistance. Chengappa et al. [2] regarded this yeast species as the cause of reproductive tract infections in mares. The occurrence of *T. ciferrii* on the skin and mucous membranes of all the mares in our study (Tables 1, 2, 3, 4) was not accompanied by any discernible health effects. It should be stressed that *T. ciferrii* was not isolated from the vaginal mucosa in any of the horse groups examined. This may indicate that *T. ciferrii* occurring on the skin and mucous membranes of the English full blood horses is an opportunistic rather than invasive fungus.

An important trait of numerous pathogenic microorganisms is their ability of biofilm formation. However, none of the strains isolated and described in this paper exhibited such properties. The presence of the yeasts that are commonly regarded as pathogenic in mares (*C. albicans*, *C. lusitaniae, C. tropicalis*) is baffling; therefore, further investigations should be undertaken to elucidate the reasons for the absence of clinical symptoms normally caused by the microflora in English full blood horses.

The results obtained suggest that a majority of the yeasts isolated may be components of normal microflora in horse organisms. Even as commensals, yeasts occurring in horses are extremely important from an epidemiological point of view. They can undergo transformation from opportunistic to virulent organisms, due to the effect of a number of environmental factors that have direct or indirect immunosuppressive action, for example viral infection or antibiotic therapy.

## Summary

The present paper presents pioneer study results. Representatives of five microflora species of the genus *Candida* and *T. ciferrii* were isolated from the skin and mucous membranes of the English full blood mares. Three species (*C. guiliermondii, C.* sp. and *T. ciferrii*) were present in all the groups of mares, irrespective of their reproduction performance. *C. albicans* and *C. lusitaniae* did not occur in the mares that had foaled. However, since the yeasts were absent from the vaginal mucosa of the mares with conception failure and barren mares, they should not be associated with reproductive disorders. The most prevalent yeast, irrespective of reproductive performance, was *C. guiliermondii*. The growth of the yeasts isolated from mares’ vaginas, compared to that of all the isolated species, was insignificant in all the groups of horses under study and ranged from 8 to 21 %. In mares that exhibited conception failure, it was almost threefold higher (21 %) than in the high-quality mares (8 %). *C. tropicalis* may be regarded as opportunistic flora in mares, as it was isolated from the vaginal mucosa of the mares that had foaled. *T. ciferrii* occurring on the skin and mucous membranes of the English full blood horses is an opportunistic rather than invasive fungus. The results obtained suggest that the yeasts isolated may constitute the normal microflora in horses’ organisms, although their contribution to reproductive disorders cannot be excluded clearly.
